# Analysis of the dose-dependent stage-specific in vitro efficacy of a multi-stage malaria vaccine candidate cocktail

**DOI:** 10.1186/s12936-016-1328-0

**Published:** 2016-05-17

**Authors:** Alexander Boes, Holger Spiegel, Robin Kastilan, Susanne Bethke, Nadja Voepel, Ivana Chudobová, Judith M. Bolscher, Koen J. Dechering, Rolf Fendel, Johannes F. Buyel, Andreas Reimann, Stefan Schillberg, Rainer Fischer

**Affiliations:** Fraunhofer Institute for Molecular Biology and Applied Ecology IME, Forckenbeckstrasse 6, 52074 Aachen, Germany; Institute for Molecular Biotechnology, RWTH Aachen University, Worringerweg 1, 52074 Aachen, Germany; TropIQ Health Science, Geert Grooteplein 28, Huispost 268, 6525 GA Nijmegen, The Netherlands

**Keywords:** Calibration-free concentration analysis (CFCA), Combination vaccine, *Pichia pastoris*, *Plasmodium falciparum*

## Abstract

**Background:**

The high incidence and mortality rate of malaria remains a serious burden for many developing countries, and a vaccine that induces durable and highly effective immune responses is, therefore, desirable. An earlier analysis of the stage-specific in vitro efficacy of a malaria vaccine candidate cocktail (VAMAX) considered the general properties of complex multi-component, multi-stage combination vaccines in rabbit immunization experiments using a hyper-immunization protocol featuring six consecutive boosts and a strong, lipopolysaccharide-based adjuvant. This follow-up study investigates the effect of antigen dose on the in vitro efficacy of the malaria vaccine cocktail using a conventional vaccination scheme (one prime and two boosts) and a human-compatible adjuvant (Alhydrogel^®^).

**Results:**

IgG purified from rabbits immunized with 0.1, 1, 10 or 50 µg doses of the VAMAX vaccine candidate cocktail was analysed for total IgG and antigen-cocktail-specific titers. An increase in cocktail-specific titers was observed between 0.1 and 1 µg and between 10 and 50 µg, whereas no significant difference in titers was observed between 1 and 10 µg. Antigen component-specific antibody titers and stage-specific in vitro efficacy assays were performed with pooled IgG from animals immunized with 1 and 50 µg of the VAMAX cocktail. Here, the component-specific antibody levels showed clear dose dependency whereas the determined stage-specific in vitro IC_50_ values (as a correlate of efficacy) were only dependent on the titer amounts of stage-specific antibodies.

**Conclusions:**

The stage-specific in vitro efficacy of the VAMAX cocktail strongly correlates with the corresponding antigen-specific titers, which for their part depend on the antigen dose, but there is no indication that the dose has an effect on the in vitro efficacy of the induced antibodies. A comparison of these results with those obtained in the previous hyper-immunization study (where higher levels of antigen-specific IgG were observed) suggests that there is a significant need to induce an immune response matching efficacy requirements, especially for a *Pf*AMA1-based blood stage vaccine, by using higher doses, better adjuvants and/or better formulations.

## Background

Malaria remains a major challenge for the healthcare systems and economies of many developing countries, especially in sub-Saharan Africa [1] and an efficient vaccine would help to address the problems associated with constantly evolving drug resistance of the parasite, *Plasmodium falciparum* [2]. Several strategies can be chosen in the context of malaria vaccine development. Vaccines can target “lower hanging fruits” such as the prevention or reduction of clinical manifestation, pregnancy-associated malaria, and malaria transmission, or they can aim for the “holy grail” of sustained strain-transcending sterile protection. While the GSK vaccine Mosquirix^®^, based on circumsporozoite protein (*Pf*CSP) [3–5], exclusively targets the pre-erythrocytic stage of *P. falciparum* to prevent the establishment of the parasite within the liver, other approaches focus on blood-stage antigens that can be found on the surface of merozoites, to induce immune responses that block the invasion of red blood cells and thereby prevent or reduce clinical episodes. A vaccine that reduces the blood-stage parasite load may also reduce transmission. In addition to *Pf*MSP1 and fragments thereof [6, 7], *Pf*MSP2 [8] and *Pf*MSP3 [9, 10], the apical membrane antigen *Pf*AMA1 [11, 12] has been proposed as a promising blood-stage vaccine candidate. This molecule is highly polymorphic, so allele-specific immune responses show moderate or even low efficacy against heterologous strains of *P. falciparum*. The allelic diversity of *Pf*AMA1 has been addressed by the design of three artificial, diversity-covering variants (DiCo1-3) [13] or by combining three [14], four [15], six [16] or seven [17] different *Pf*AMA1 alleles. In the field of transmission-blocking vaccines (TBVs), a zygote and ookinete surface antigen remains the leading candidate [18] and is undergoing clinical investigation [19]. The authors of the current article previously designed and analysed the stage-specific in vitro efficacy of a malaria antigen cocktail [20] comprising three recombinant fusion proteins combining the three *Pf*AMA1_DiCo variants each with *Pfs*25, and *Pf*MSP1-19 (VAMAX1), *Pf*CSP_TSR (VAMAX2) or *Pf*CelTOS (VAMAX4). The latter work demonstrated the ability of the multi-component vaccine cocktail (VAMAX1, 2 and 4) featuring antigens from different stages of the *P.falciparum* life cycle to elicit parasite growth-inhibitory responses against the pre-erythrocytic stage, the blood stage and the sexual stage. However, the authors observed the proteolytic degradation of VAMAX4, leading to the loss of the C-terminal fusion partner *Pf*CelTOS. Modified variants of VAMAX4 were, therefore, investigated, and in VAMAX6 *Pf*CelTOS was replaced with a promising epitope (Q5A) [21] derived from the blood-stage antigen *Pf*RH5 [22]. In contrast to the immunization experiments with the initial cocktail (VAMAX1, 2 and 4) which featured a hyper-immunization regime (high 200 µg prime dose and five boosts of 100 µg), the work described herein investigated the dose-dependency of immune responses and stage-specific in vitro efficacy for the modified cocktail (VAMAX1, 2 and 6) using a human-compatible adjuvant (Alhydrogel®) and a one prime/two boosts vaccination regime. Additionally, the authors sought to confirm the observed correlation between antigen abundance within the improved VAMAX cocktail and the proportions of the corresponding antigen-specific antibodies found in the rabbit immune IgG fraction.

## Methods

### Construct design and cloning

**Fig. 1:**
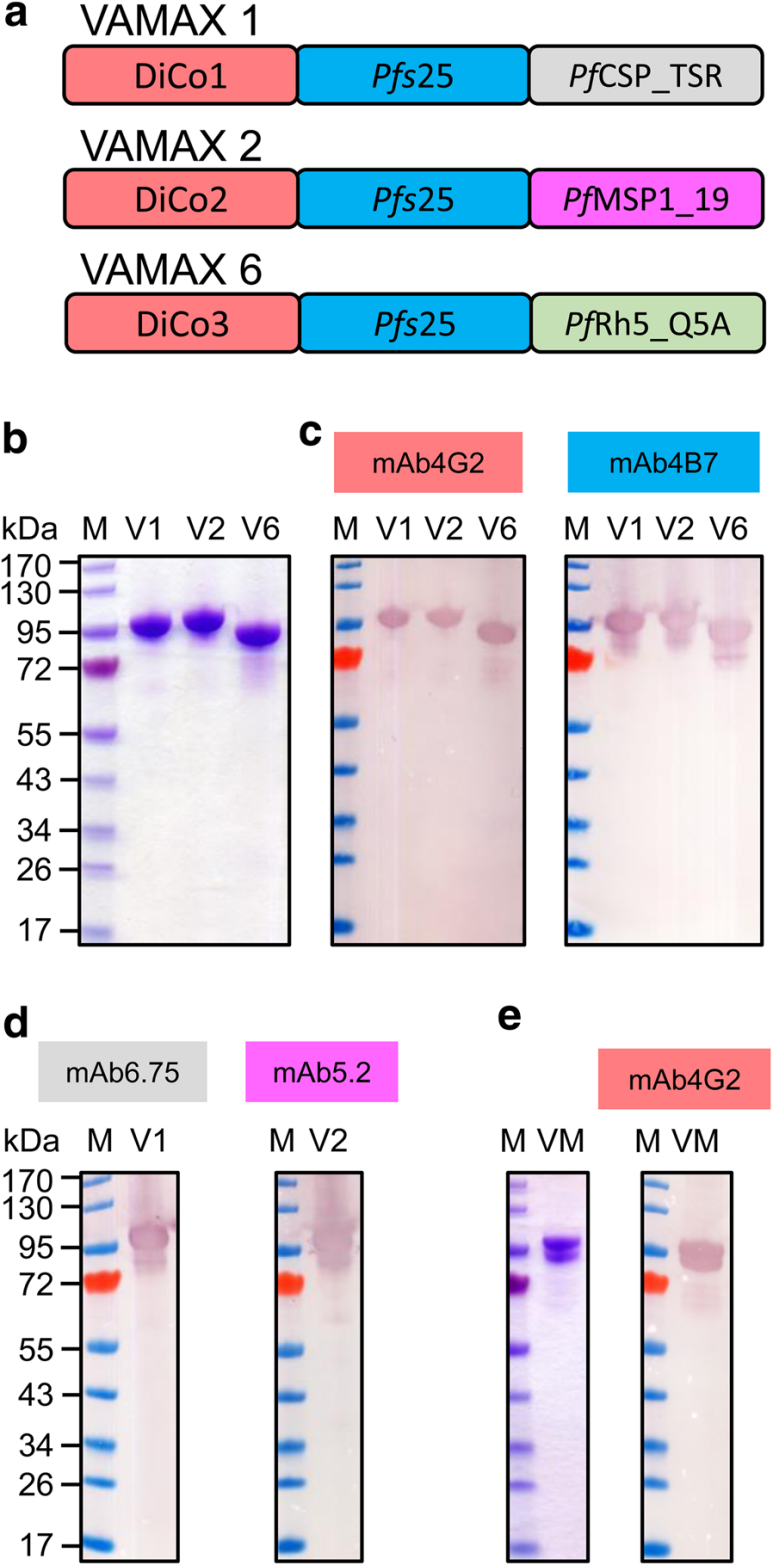
Antigen design, LDS-PAGE and immunoblot blot analysis of VAMAX 1, VAMAX 2 and VAMAX 6 expression. **a** Schematic representation of the VAMAX 1, VAMAX 2 and VAMAX 6 fusion antigens. *DiCo1–3* diversity covering variants of *Pf*AMA1 (*red*); *Pfs25* *P. falciparum* 3D7 sexual-stage surface antigen *Pfs*25 (*blue*); Pf*CSP_TSR* thrombospondin-related region from *P. falciparum* 3D7 circumsporozoite protein *Pf*CSP (*gray*); *PfMSP1_19* C-terminal 19 kDa fragment of merozoite surface protein 1 (*Pf*MSP1_19) from *P. falciparum* strain FUP (*pink*); *PfRH5_Q5A* Short amino acid stretch of the reticulocyte-binding protein homologue 5 (*Pf*RH5) of *P. falciparum* 3D7, epitope of the invasion inhibitory antibody mAb Q5A. **b** LDS-PAGE analysis under non-reducing conditions of VAMAX 1 (V1), VAMAX 2 (V2) and VAMAX6 (V 6) after purification (3.4 µg of each VAMAX fusion protein per* lane*). **c** Immunoblot analysis under non-reducing conditions of purified VAMAX fusion proteins (V1, V2 and V6): 1.3 µg of each VAMAX fusion protein was loaded and the fusion proteins (V1, V2 and V6) were detected either with the plant-derived chimeric *Pf*AMA1-specific mAb4G2 (*left panel*) or the murine *Pfs*25-specific mAb4B7 (MRA28, MR4, ATCC) (*right panel*). **d** Immunoblot analysis under non-reducing conditions. *Left panel*: Purified VAMAX 1 (V1, 1.3 µg) was detected using a *Pf*CSP_TSR domain-specific murine monoclonal antibody mAb6.75; *right panel*: Purified VAMAX 2 (V2, 1.3 µg) was detected using the *Pf*MSP1_19-specific murine monoclonal antibody mAb5.2 (MRA94, MR4, ATCC). **e** LDS-PAGE (*left panel*, 3.4 µg) and immunoblot analysis (*right panel*, 1.3 µg) of an equimolar mixture of VAMAX 1, VAMAX 2 and VAMAX 6 (VM). Immunoblot analysis was performed under non reducing conditions and the equimolar VAMAX mixture was detected with the plant-derived chimeric *Pf*AMA1-specific mAb4G2. Primary antibodies were detected with alkaline phosphatase-labeled goat anti-human or anti-mouse antisera. M: PageRuler™ Pre-stained protein ladder (Fermentas)

The design and cloning of constructs VAMAXl and VAMAX2 and selection of recombinant *Pichia pastoris* clones was previously described [20]. VAMAX6 comprising *Pf*DiCo3, *Pfs*25 and the epitope of blood-stage antigen RH5-specific, parasite-inhibitory monoclonal antibody Q5A (amino acids 198–213, GeneID PF3D7_0424100) was obtained as a *Pichia pastoris* codon-optimized synthetic gene from GeneArt (Invitrogen, Carlsbad, CA) (Fig. 1a). The construct was inserted as previously described [20] into a *Pichia pastoris* expression vector containing the methanol inducible AOX1 promoter and terminator to control transgene expression. Cloning was confirmed by DNA sequencing. The constructs did not contain any potential N-glycosylation motif which occur in the natural sequences of *Pf*AMA1, and *Pfs*25. For details on the used knock-out mutations refer to the following publications [23, 24].

### Transformation and screening of *Pichia pastoris*

The transformation, cultivation and screening of *Pichia pastoris* strain CBS704 was carried out as previously described [25].

### Fed-batch fermentation and purification of the antigens

The pre-cultures were prepared and the cultivations were carried out as previously described [20, 25] with minor changes. The number of fermentation phases was reduced to two, so the process consisted only of a batch and an immediate induction phase. For the latter phase, the temperature was lowered to 25 °C and the methanol concentration was kept constant at 0.25 % (v/v) by the use of an ALKOSENS probe combined with an ACETOMAT NII controller (Heinrich Frings GmbH & Co. KG, Bonn, Germany). During induction, the dissolved oxygen tension continuously dropped to 0 % as the stirrer speed reached a maximum of 600 rpm. When a total of 2.7 kg methanol was added, the pH was adjusted to 7.0 followed by the harvest and centrifugation of the broth (9000×*g*, 20 min, 4 °C). The culture supernatant was collected for immediate processing or for storage at–20 °C. Antigens were purified by immobilized metal ion affinity chromatography (IMAC) using Chelating Sepharose Fast Flow (GE Healthcare Life Sciences, Little Chalfont, UK) as a capture step (the N-terminal pro-peptide of *Pf*AMA1 included in all three constructs binds to copper-charged chelating Sepharose Fast Flow resin) followed by buffer exchange using a HiPrep 26/10 column (GE Healthcare) and anion exchange (AEX) chromatography using Q Sepharose HP (GE Healthcare). Finally, samples were concentrated with a Centriprep YM-10 concentrator (Merck, Darmstadt, Germany) and polished by size exclusion chromatography (SEC) on a Sephacryl S-100 HR 16/60 column (GE Healthcare).

### LDS-PAGE and immunoblot analysis

Purified VAMAX1, VAMAX2 and VAMAX6 were fractionated separately under non-reducing conditions on 4–12 % (w/v) polyacrylamide gradient gels (NuPage, Thermo Fisher Scientific, Waltham, MA, USA) and either stained with Coomassie Brilliant Blue (Fig. 1b) or transferred onto a nitrocellulose membrane (Whatman, GE Healthcare) for immunoblot analysis as previously described [26]. After blocking with 5 % (w/v) skimmed milk dissolved in phosphate buffered saline (PBS), the VAMAX1, 2 and 6 proteins were probed with the *Pfs*25-specifc monoclonal antibody mAb4B7 (obtained by MR4) as well as the plant-derived chimeric variant *Pf*AMA1-specific mAb4G2 (provided by Stefan Menzel, Fraunhofer IME, Aachen, Germany) (Fig. 1c). Additionally, mAb6.75, a *Pf*CSP_TSR-specific murine monoclonal antibody generated by Christoph Kühn (Fraunhofer IME), was used specifically to detect the C-terminal *Pf*CSP of VAMAX1 and the murine mAb5.2 (obtained by MR4) enabled the specific detection of the C-terminal *Pf*Msp1_19 of VAMAX2. All primary antibodies were used at a concentration of 1 µg/ml (Fig. 1d). The secondary antibody was an alkaline phosphatase-labeled goat anti-mouse H + L or alkaline phosphatase-labeled goat anti-human Fc (both from Jackson Immunoresearch, West Grove, PA, USA).

### Mass spectrometry (MS)

Proteins fixed in polyacrylamide gels were reduced, alkylated and digested with trypsin (Promega, Mannheim, Germany) as previously described [27]. The resulting peptides were analysed by nanoHPLC (UltiMate 3000 HPLC system, LC Packing, Dionex, Idstein, Germany) coupled to an amaZon ETD MS ion trap spectrometer (Bruker Daltonics, Bremen, Germany) using ESI nano sprayer. MS/MS spectra were searched for peptides of interest using the local search engine Mascot Search v2.3.01 (Matrix Science Ltd, London, UK). Specific information on the MS measurements was previously reported [20].

### Antigen formulation

Each purified VAMAX fusion protein (VAMAX 1, VAMAX 2 and VAMAX 6) was dialyzed against the formulation buffer (2.4 mM NaH_2_PO_4_, 2.6 mM K_2_HPO_4_, 0.125 mM Na_2_EDTA, 0.27 M D(-)mannitol, pH 6.8) (slightly modified from [28]). An equimolar VAMAX mixture was prepared and filter sterilized using a Pall Acrodisc 32 mm Supor filter (pore size 0.2 µm). The equimolar VAMAX mixture was adjusted to 200 µg/ml (50 µg vaccine dose), 40 µg/ml (10 µg vaccine dose), 4 µg/ml (1 µg vaccine dose) or 0.4 µg/ml (0.1 µg vaccine dose) with filter-sterilized formulation buffer and 1.7 ml of each VAMAX mixture was filled into clear glass injection vials (2 ml, 13-mm crimp neck) and lyophilized. The vials were sealed with rubber stoppers and aluminum crimp seals. One vial was used per immunization time point and dose. For the alum formulation, a pre-dilution of GMP-grade Alhydrogel® was prepared by mixing 3.2 ml Alhydrogel® with 6.8 ml saline under sterile conditions. The Alydrogel® pre-dilution was mixed by gentle agitation and allowed to equilibrate for 60 min at 4 °C. A lyophilized vial was reconstituted with 1.7 ml saline under sterile conditions and four aliquots of 280 µl were transferred to sterile 1.5-ml reaction tubes. Samples were stored at 4 °C, and 280 µl of the Alhydrogel® pre-dilution was added to each vial to obtain the final immunization dose, resulting in 0.8 mg Al^3+^ per 500 µL immunization dose. The adsorption of the VAMAX mixture to Alhydrogel® under formulation conditions was determined to be >95 %.

### Rabbit immunization, titer determination

Rabbits were housed, immunized and sampled by Biogenes GmbH (Berlin, Germany), according to national animal welfare regulations. After equilibration of the vials for 60 min at 4 °C, each rabbit was immunized with an injection dose of 500 µl. For each dose (0.1, 1, 10 and 50 µg), four rabbits were immunized on days 0 (prime), 28 (boost 1) and 56 (boost 2). Serum samples were taken on days 0 (pre-immune) and 70 (final bleed). Titer determination was carried out as previously described by direct ELISA using the VAMAX antigen cocktail to coat the ELISA plates (100 ng/well) [26].

### Antibody purification

Total IgG was purified by conventional Protein A affinity chromatography using 40 ml immune serum (day 70) from rabbits immunized with the 1-µg dose and 20 ml immune serum from rabbits immunized with the 50-µg dose. Bound antibodies were eluted with 100 mM glycine (pH 3.0) and directly neutralized by adding 10 % (v/v) 1 M Tris–HCl (pH 8.0). The elution fractions were concentrated to 2.5 ml using VIVASPIN 15R centrifugal concentration devices with a molecular weight cut off of 30 kDa (Sartorius, Göttingen, Germany), and exchanged against 5 mM phosphate buffer (pH 7.5) using disposable PD10 columns (GE Healthcare). After lyophilization, the samples were reconstituted in 600 µl PBS and filter sterilized (pore size 0.2 µm). Antibody integrity and the total IgG concentration were determined by analytical gel filtration as previously described [29]. The final IgG preparations were used for calibration-free concentration analysis (CFCA) and all subsequent in vitro inhibition assays.

### Calibration-free concentration analysis

The antigen-specific antibody concentrations were measured in the purified antibody preparations by CFCA [30, 31] using a Biacore T200 instrument. The purified antigens comprised *Pf*AMA1 variants DiCo 1–3 (provided by Stephan Hellwig, Fraunhofer IME, with kind permission from Bart Faber, BPRC, The Netherlands), *Pf*CSP_TSR (in the context of CCT, a fusion protein containing *Pf*CSP_TSR, *Pf*CelTOS and *Pf*TRAP_TSR [32]), *Pfs*25 (in the context of F0, a fusion protein containing *Pfs*25gk and *Pfs*230_C0, [24]) and *Pf*MSP1_19 (in the context of a tetravalent *Pf*Msp1-19 fusion protein, provided by Güven Edgü, Fraunhofer IME). The antigens were separately covalently coupled to CM5-S-Series sensor chips by standard EDC-NHS chemistry as previously reported [26]. To match the assay specifications in terms of the initial binding rates at 5 µl/min, purified rabbit immune IgG was used at a concentration of 20–100 µg/ml as determined by test injections. Initial binding rates at 5, 30 and 100 µl/min were used to calculate antigen-specific IgG concentrations using the CFCA tool in the Biacore T200 evaluation software.

### Assays

#### Sporozoite gliding motility assay

*Plasmodium falciparum* NF54 sporozoites were isolated and used in an adapted sporozoite gliding motility (SGM) assay [33]. Each triplicate assay required 10,000 sporozoites per well in a 96-well glass bottom black plate. After 90 min incubation at 37 °C, 98 % relative humidity, 93 % (v/v) N_2_, 4 % (v/v) CO_2_ and 3 % (v/v) O_2_, gliding trails were washed and stained with biotinylated anti-CSP monoclonal antibody 3SP2 followed by AlexaFluor594-labeled streptavidin (Invitrogen). An automated high-content microscope (Leica) was used to capture nine images per well at 1000× magnification. Images were automatically processed with FIJI imaging software, as described elsewhere (Bolscher et al., pers. comm.).

#### Sporozoite invasion and liver stage development assay

The sporozoite invasion and liver stage development (SILSD) assay [29] was adapted as follows. Cryopreserved human hepatocytes (Tebu-Bio) were thawed and cultured in a 96-well clear bottom black plate (BD Falcon) for 2 days according to manufacturer’s protocol. Per well, 50,000 *Pf* NF54 sporozoites were pre-incubated with purified rabbit IgG samples for 30 min on ice, after which the mixture was transferred onto the hepatocytes. Samples were tested in triplicate and the medium was refreshed daily according to the manufacturer’s protocol. On day 6 after sporozoite invasion, hepatocytes were washed and fixed with 4 % (v/v) paraformaldehyde. Vehicle and 3SP2 control wells were stained with rabbit anti-*Pf*HSP70, followed by AlexaFluor594-labeled goat anti-rabbit IgG and 4′,6-diamidino-2- phenylindole (DAPI). Vehicle and rabbit IgG samples were stained with a pool of mouse monoclonal antibodies directed against *Pf*Bip (binding protein, a marker of the endoplasmic reticulum), *Pf*EXP-1 (exported antigen AG 5.1), *Pf*HSP70 (heat shock protein 70) and *Pf*MSP1 (merozoite-specific protein 1) followed by AlexaFluor488-labeled chicken anti-mouse IgG and DAPI. To determine the number of positively-stained (infected) hepatocytes, an automated high-content microscope (Leica) was used to capture 25 images per well at 100× magnification. Images were automatically processed with FUJI imaging software, as described elsewhere (Bolscher et al., pers. comm.).

#### In vitro growth inhibition assay

The ability of purified polyclonal rabbit IgGs to inhibit the growth of *P. falciparum* strains 3D7, 7G8 and V1-S (obtained through the MR4) was determined using growth inhibition assays (GIAs) as previously described [34]. Reversal of growth inhibition assays were carried out as previously described [29].

#### Standard membrane feeding assay

The standard membrane feeding assay (SMFA) [35] was adapted as follows. The diluted rabbit IgG sample (36 μl) was mixed with 300 μl *Pf*NF54:HSP70:GFP:luc gametocyte culture adjusted to a final haematocrit of 44.7 % red blood cells in 44.6 % human serum-containing active complement. The mixture was fed to *Anopheles stephensi* mosquitoes. After 8 days, the mosquitoes were frozen at –20 °C and the next day 24 mosquitoes per cage were analysed for luciferase expression as a measure for oocyst intensities [36]. As controls, 24 uninfected mosquitoes were analysed to determine background luminescence levels.

### Statistics

The antibody titers determined by CFCA were averaged (n = 4) for each combination of antibody type (VAMAX mix titer, total IgG titer, domain-specific titers) and sample group [antigen dose, pre-immune serum (P) and immune serum (I)] and the normal distribution of the data in each of these sample sets was tested using the Kolmogorow–Smirnow test (a = 0.05) [37]. The corrected standard deviation of each sample set was used to calculate its variance. Variances were compared using a pairwise F-test between sample groups for each antibody type (a = 0.05). If differences between the variances were not significant and the data points were normally distributed, a two-sided *t* test (a = 0.05) was used to identify significant differences between the antibody titers of sample set pairs. In case multiple group comparisons were made, Šídák’s multiple comparisons post hoc test was used following analysis of variance (ANOVA). For the VAMAX mix titers, the data were normalized by log-transformation for analysis. The association between the relative proportion of component-specific IgG and the relative proportion of molecular weight calculated for the components was analysed by calculating the Spearman’s rank correlation coefficient. The IC_50_-values were estimated by non-linear regression using the following model:

$${\text{Y}} = 100/( 1+10^{ \wedge } ( {\text{LogIC}}_{50} - {\text{X}} ))$$

### Ethics approval and consent to participate

The human cells used in this study were provided by Human HepCell (Paris, France). The company obtained the permission, issued by the French Ministry of Education and Research Ethical Commitee (Decision n° AC 2011-1308 de la Cellule Bioéthique de la Direction Générale pour la Recherche et l´Innovation du novembre 2011) to provide such samples for research use. The donors were informed by their surgeon that the tissues collected during the surgery could be used for research purposes only and that they get no financial compensation for this donation. For each donor, a consent of non-opposition is signed.

Rabbits were housed, immunized and sampled by Biogenes GmbH (Berlin, Germany), according to national animal welfare regulations. The animal facilities and protocols were reviewed and approved by: Landesamt für Landwirtschaft, Lebensmittelsicherheit und Fischerei MecklenburgVorpommern (LALLF M-V) (Approval No: 7221.3-2-030-13). To isolate the blood after immunization according to national regulations the animals were anesthetized using Ventranquil, stunned using a captive bolt device and exsanguinated by throat cut.

## Results

### Production of the antigen cocktail

Recombinant *Pichia pastoris* cultures individually transformed with the three expression constructs (VAMAX 1, 2 and 6, see Fig. 1a) were used for fed-batch fermentation (15-L scale). After purification by IMAC and SEC, the purity and integrity of the recombinant proteins was analysed by LDS-PAGE followed by Coomassie staining, immunoblotting and analytical SEC. Monoclonal antibodies were available for the specific detection of each protein domain within the three VAMAX fusion proteins except the *Pf*RH5 Q5A epitope. The proteins were obtained at high purity (>95 % determined by analytical SEC) the apparent molecular masses agreed with the calculated values of 87,592 Da for VAMAX 1 (measured 87,463 Da), 89,906 Da for VAMAX 2 (measured 90,312 Da) and 80,864 Da for VAMAX 6 (measured 80,813 Da) (Table 1), there was no indication of relevant amounts of degradation products, and the specific monoclonal antibodies detected the corresponding antigen domains (Fig. 1b–e). The yields after purification were 10, 13 and 4 µg/ml (calculated after purification and given as µg fusion protein/ml culture supernatant) for VAMAX 1, VAMAX 2 and VAMAX 6, respectively. MALDI-TOF–MS was used to determine the identity and integrity of the three recombinant proteins by analysing the tryptic fragments and calculating the total mass. In accordance with the immunoblot data, tryptic fragments representing each of the protein domains were identified. Together with the total mass analysis (Table 1), where approximately the expected molecular masses were observed for the three recombinant fusion proteins (−0.2 % for VAMAX 1, +0.4 % for VAMAX 2 and −0.1 % for VAMAX 6) these results confirmed the presence of fully intact fusion proteins.

**Table 1:** Calculated and measured molecular weight of the VAMAX variants

Sample	Theoretical mass [Da]	Measured mass [Da]	Discrepancy [%]
VAMAX1	87,592	87,463	−0.2
VAMAX2	89,906	90,312	+0.4
VAMAX6	80,864	80,813	−0.1

### Immunization, determination of titers and total IgG levels

**Fig. 2:**
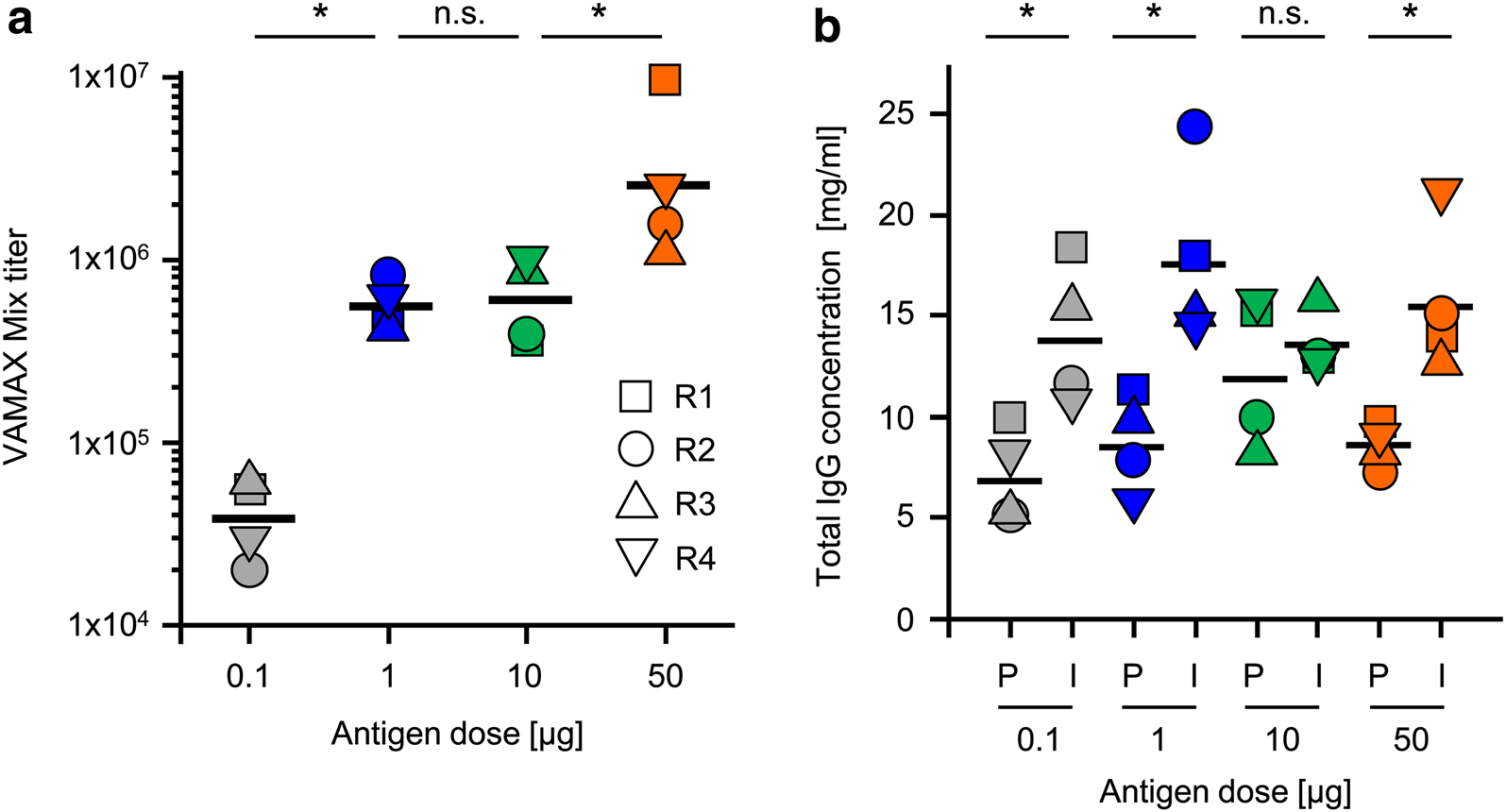
Determination of the VAMAX-mix specific titer and quantification of total IgG concentration in serum samples. **a** The VAMAX-mix-specific titer after immunization of four rabbits in each antigen group (0.1, 1, 10 and 50 µg of VAMAX-mix formulated with Alhydrogel® using a one prime/two boost immunization schedule) were assessed by direct coating ELISA using the VAMAX-mix as the coating antigen. **b** Total IgG concentration in the pre-immune (P) and immune serum (I) collected on day 70 was quantified by surface plasmon resonance spectroscopy using a protein A capture chip and a quantified rabbit IgG reference for the generation of a standard curve. *Gray*: 0.1 µg antigen dose; *blue*: 1 µg antigen dose; *green*: 10 µg antigen dose; *orange*: 50 µg antigen dose; *square*: Rabbit 1 (R1); *circle*: Rabbit (R2); *upward triangle*: Rabbit 3 (R3); *downward triangle*: Rabbit (R4); *black horizontal line*: geometric mean. For detailed statistical analysis refer to “Methods” section. *Asterisks* indicate a significant difference (**a** = 0.05), whereas “*NS*” indicates no significant differences

Immune sera from rabbits immunized with the different doses of the VAMAX 1, 2 and 6 cocktail were used for the determination of cocktail-specific titers. Figure 2a shows the titers of individual animals as well as the geometric mean obtained after immunization with the corresponding doses. Comparing the geometric means of the four animals per group, a greater than tenfold increase in titer (from 3.8 × 10^4^ to 5.6 × 10^5^) was observed between the 0.1 and 1 µg doses, which was statistically significant. Only a marginal difference in titer was observed between the 1 and 10 µg antigen doses (from 5.6 to 6.0 × 10^5^) but the titers showed another statistically significant increase between the 10 and 50 µg doses (from 6.0 × 10^5^ to 2.5 × 10^6^). The observed titers led to the selection of samples from the 1 and 50 µg doses for pooling (hereafter P1 and P50, respectively) and subsequent analysis in the stage-specific parasite inhibition assays. Additionally, the total IgG in the sera of the animals before (P) and after immunization (I) were compared. As shown in Fig. 2b, the total IgG levels after immunization were independent of the antigen dose because there were no statistically significant differences between the geometric mean IgG levels between immune sera of the different dose groups, which all contained total IgG concentrations of ~14 mg/ml.

### Determination antigen-specific IgG levels

**Fig. 3:**
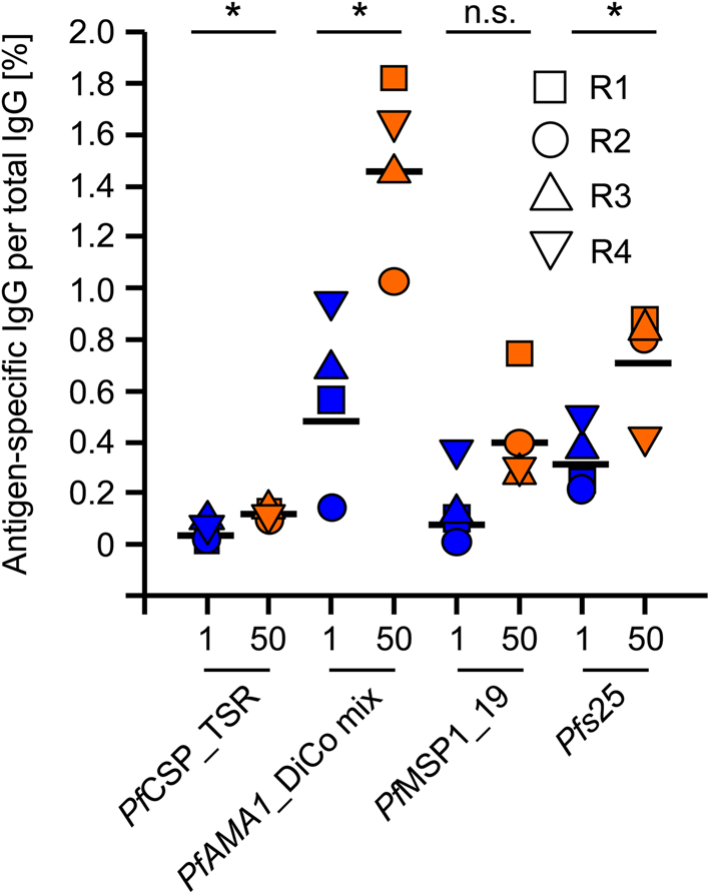
Antigen-specific IgG concentration in the purified total IgG fraction. The antigen specific IgG concentration for the corresponding antigen (*Pf*CSP_TSR, *Pf*AMA1_DiCo mix, *Pf*MSP1_19 and *Pfs*25) was quantified in the total IgG fraction purified from the immune serum samples collected on day 70 from rabbits receiving the 1 µg (P1, *blue*) and 50 µg antigen dose (P50, *orange*). The quantification was performed using the calibration-free concentration analysis module of the Biacore T200 instrument (surface plasmon resonance spectroscopy) and the antigen-specific IgG concentration is expressed as a percentage of total IgG. Square: Rabbit 1 (R1); *circle*: Rabbit (R2); *upward triangle*: Rabbit 3 (R3); *downward triangle*: Rabbit (R4); *black horizontal line*: geometric mean. For detailed statistical analysis refer to the “Methods” section. *Asterisks* indicate a significant difference (**a** = 0.05), whereas “*NS*” indicates no significant differences

CFCA was used to determine the amounts of antigen-specific IgG within pools of purified total IgG from immune sera in the P1 and P50 dose groups, enabling the correlation of inhibition values obtained from stage-specific in vitro assays not only with total IgG but also with antigen and/or stage-specific IgG concentrations (Table 2). The highest concentration of antigen-specific IgG was measured for the *Pf*AMA1_DiCo mix, followed by *Pfs*25, *Pf*MSP1-19 and *Pf*CSP_TSR. Q5A-specific IgG could not be quantified since no Q5A peptide-specific reactivity could be detected in rabbit immune IgG by ELISA. The values (µg/ml antigen-specific IgG) were used to calculate percent values for the specificity of each antigen in relation to total IgG (Fig. 3). Here, pronounced differences (up to three-fold for the *Pf*AMA1–DiCo mix) were observed between the P1 and P50 groups for all four antigens. The observed differences between the P1 and P50 groups were statistically significant with the exception of *Pf*MSP1_19, although even in this case there was a slight tendency towards higher concentrations of *Pf*MSP1_19-specific antibodies in the P50 group.

**Table 2:** Total and component-specific IgG concentrations in the purified rabbit immune IgG preparations

Dose [µg]	Rabbit	Total IgG [mg/ml]	VAMAX-specific IgG [mg/ml]	*Pf*CSP_TSR-specific IgG [mg/ml]	*Pf*AMA1_DiCo mix-specific IgG [mg/ml]	*Pf*MSP1_19-specific IgG [mg/ml]	*Pfs*25-specific IgG [mg/ml]
50	R1	148.1	5.290	0.190	2.700	1.100	1.300
50	R2	136.4	3.170	0.130	1.400	0.540	1.100
50	R3	130.9	3.540	0.180	1.900	0.360	1.100
50	R4	127.1	3.160	0.150	2.100	0.380	0.530
1	R1	157.9	1.466	0.026	0.890	0.160	0.390
1	R2	125.6	0.481	0.021	0.180	0.010	0.270
1	R3	160.0	2.040	0.150	1.100	0.190	0.600
1	R4	116.0	2.188	0.083	1.100	0.425	0.580

### In vitro efficacy assays

The in vitro efficacy of the different doses of the VAMAX vaccine cocktail were compared using appropriate stage-specific in vitro parasite inhibition assays: pre-erythrocytic/liver stage (SGM and SILSD assays), blood stage (GIAs), and sexual stage (SMFAs). The assays were carried out using purified and pooled immune IgG preparations from the P1 and P50 dose groups.

### Liver stage efficacy

**Fig. 4:**
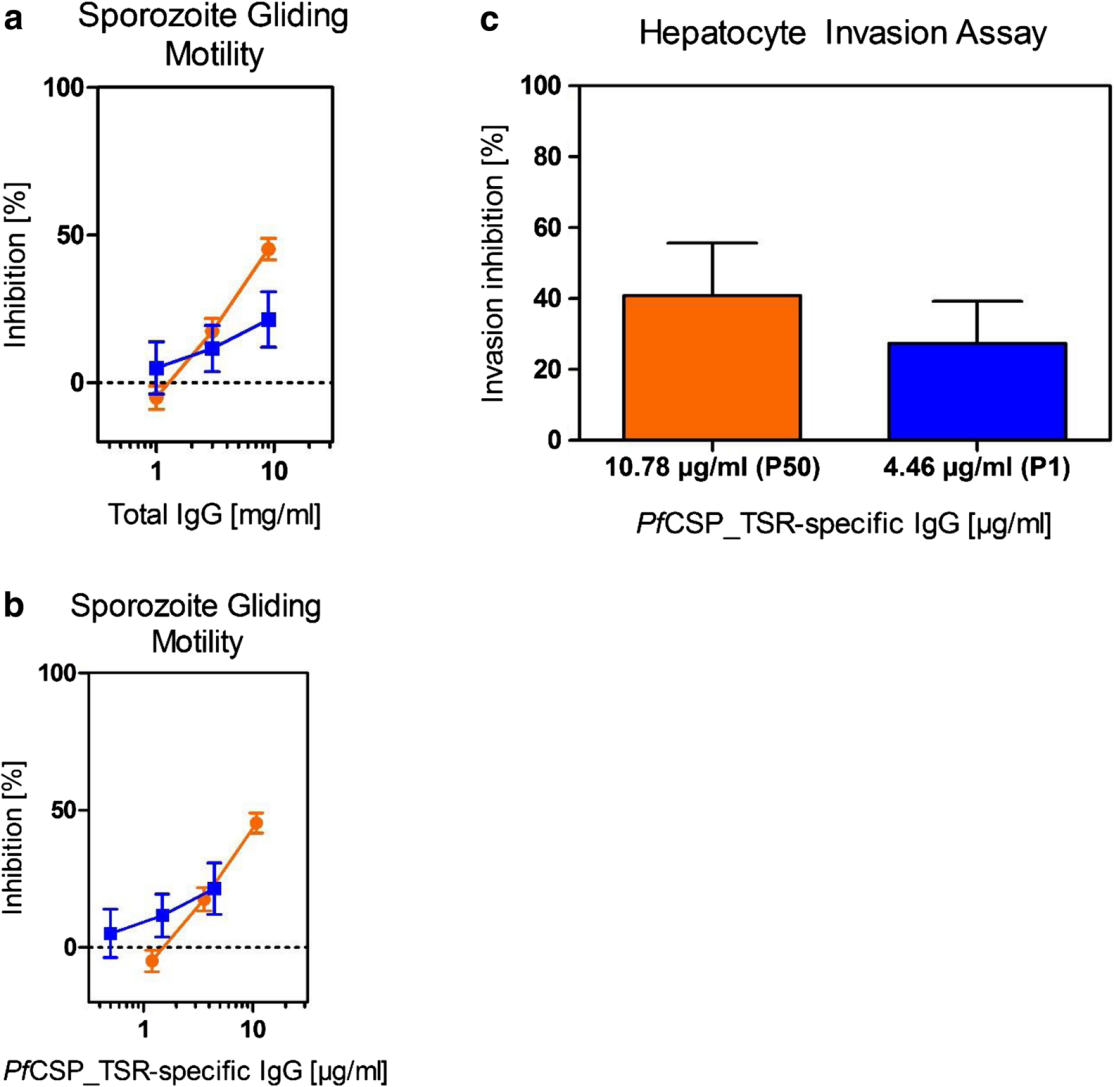
Confirmation of pre-erythrocytic stage in vitro efficacy by sporozoite gliding motility (SGM) and sporozoite invasion and liver stage development (SILSD) assays. The in vitro efficacy of antibodies induced by VAMAX-mix immunization was assessed by SGM and SILSD assays. Total IgG was purified from rabbit immune serum samples collected on day 70 and the total IgG fractions from the corresponding groups (four rabbits per group) were pooled (P1: 1 µg antigen dose, *blue*; P50: 50 µg antigen dose, *orange*). Data represent means and standard deviations of three experiments. **a** The dose-dependency of sporozoite gliding motility inhibition was investigated for P1 and P50 using 9, 3 and 1 mg/ml total IgG (**a**) and the inhibition was also correlated with the *Pf*CSP_TSR-specific antibody concentration (**b**). Based on both* graphs*, the IC_50_ values for total IgG as well as *Pf*CSP_TSR-specific antibodies were calculated and summarized in Table 3. **c** The inhibition of sporozoite invasion and liver-stage development was assessed for P1 and P50 at 9 mg/ml total IgG and the corresponding *Pf*CSP_TSR-specific antibody concentration is illustrated

The SGM assay was carried out at concentrations of 9, 3 and 1 mg/ml total IgG. Inhibition was measured as a percentage relative to the concentration of neutral rabbit serum and was plotted against total IgG (Fig. 4a) and the corresponding *Pf*CSP-specific IgG (Fig. 4b). Because the inhibition values did not exceed 50 % even at the highest IgG concentration, the IC_50_ values were estimated for total IgG as well as *Pf*CSP-specific IgG and summarized in Table 3. An inhibition value of ±50 % was observed for the highest total IgG concentration in the case of the P50 sample, whereas only ±20 % inhibition was observed at the same total IgG concentration for the P1 sample, reflecting the quantitative differences in *Pf*CSP-specific IgG between the two dose groups (Fig. 3).

**Table 3:** Summary of IC_50_-values

SGM assay	Strain	P1	P50
		3D7	
IC_50_ [mg/ml]	Total IgG	>10.0	9.95
IC_50_ [mg/ml]	95 % CI	ND	7.625–12.98
IC_50_ [µg/ml]	*Pf*CSP_TSR-specific	>4.47	11.92
IC_50_ [µg/ml]	95 % CI	ND	9.130–15.56

GIA	Strain	P1			P50		
		3D7A	7G8	V1-S	3D7A	7G8	V1-S
IC_50_ [mg/ml]	Total IgG	27.32	31.46	23.55	8.085	8.105	9.36
IC_50_ [mg/ml]	95 % CI	18.89–39.50	24.45–40.49	17.92–30.94	5.812–11.25	7.120–9.227	7.672–11.420
		3D7	7G8	V1-S	3D7	7G8	V1-S
IC_50_ [µg/ml]	*Pf*AMA1_DiCo mix specific	160.0	184.3	137.9	120.3	120.6	139.3
IC_50_ [µg/ml]	95 % CI	110.6–231.3	143.2–237.1	105.0–181.2	86.49–167.3	106.0–137.2	114.2–169.8

SMFA	Strain	P1	P50
		NF54:HSP70:GFP:luc	
IC_50_ [mg/ml]	Total IgG	0.7879	0.1427
IC_50_ [mg/ml]	95 % CI	0.434–1.43	0.09459–0.2153
IC_50_ [µg/ml]	*Pfs*25-specific	1.409	1.049
IC_50_ [µg/ml]	95 % CI	0.6935–2.862	0.6952–1.583

Additionally, the SILSD assay was carried out by combining sporozoites with 9 mg/ml total IgG onto cryopreserved primary human liver cells. In agreement with the SGM assay, different degrees of inhibition were observed (±40 % for P50 and ±30 % for P1), which again correlated with the quantitative differences in *Pf*CSP-specific antibodies in the total IgG preparations of P1 and P50 (Fig. 4c).

### Blood stage efficacy

**Fig. 5:**
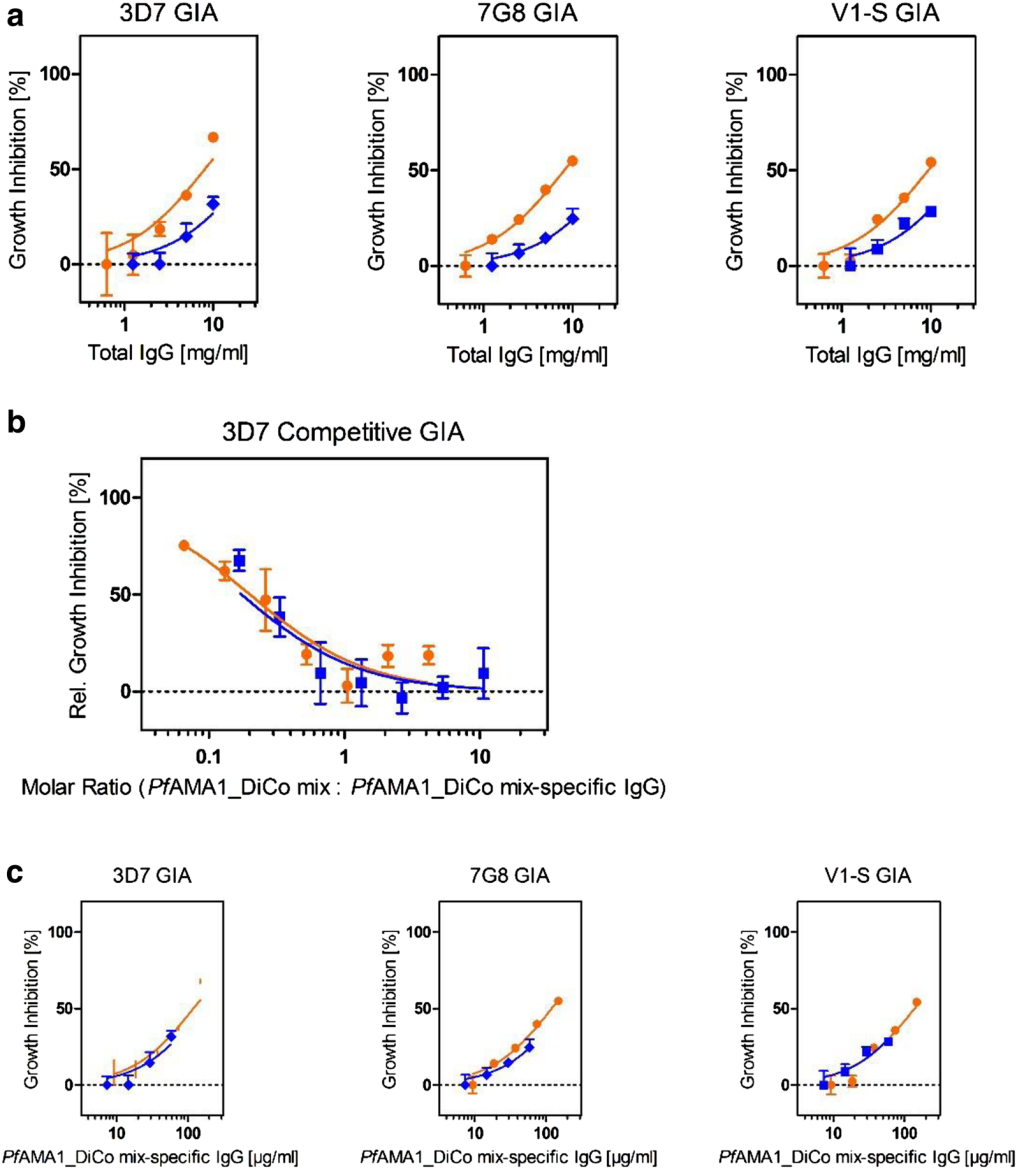
Confirmation of blood-stage in vitro efficacy measured in growth inhibition assay (GIAs) using three *P. falciparum* strains. GIAs with asexual *P. falciparum* parasites (strains: 3D7A, 7G8, V1-S) were carried out using purified total rabbit IgG pools from immune serum samples (P1: 1 µg antigen dose, *blue*; P50: 50 µg antigen dose, *orange*). Each data point represents the mean, including standard deviations of technical triplicates within one experiment. **a** The dose-dependency of GIA was investigated for P1 and P50 using 10, 5, 2.5 and 1.25 mg/ml total IgG. **b** Reversal of growth inhibition was analysed using 10 mg/ml total IgG of P1 and P50 and a serial 1/3 dilution of *Pf*AMA1_DiCo mix (250–0.34 µg/ml) as a competitor, and based on these results the inhibition was also correlated with the *Pf*AMA1_DiCo mix-specific antibody concentration (**c**). Based on both* graphs*, the IC_50_ values for total IgG as well as *Pf*AMA1_DiCo Mix-specific antibodies were calculated and summarized in Table 3

Strain-dependent blood-stage-specific efficacy was addressed by carrying out GIAs using different *P. falciparum* strains (3D7, 7G8 and V1-S) to account for the major polymorphisms covered by the three DiCo variants. Figure 5a shows the strain-specific dose-dependency of growth inhibition for total immune IgG pools P1 and P50. The different IC_50_ values are summarized in Table 3. Additionally, a reversal of growth inhibition assay was carried out to investigate the contribution of *Pf*AMA1_DiCo mix-specific IgG to blood stage-specific in vitro efficacy, using the 3D7 reference strain and *Pf*AMA1_DiCo mix as a competitor. As shown in Fig. 5b the growth-inhibitory effect of rabbit immune IgG was completely neutralized when a mixture of *Pf*AMA1_DiCo was used at equal or higher molar concentrations, compared to rabbit immune IgG antigen-binding sites. Because this finding indicates that the parasite growth-inhibitory activity measured in this assay is exclusively mediated by *Pf*AMA1_DiCo mix-specific antibodies, it was possible to estimate IC_50_ values in relation to the concentrations of *Pf*AMA1_DiCo mix-specific antibodies (Fig. 5c). The calculated IC_50_ values in relation to *Pf*AMA1_DiCo mix-specific antibodies as well as total IgG are shown in Table 3. No significant differences of IC_50_ values were observed for the *Pf*AMA1_DiCo mix-specific antibodies (120–180 µg/ml) or total IgG (8–30 mg/ml) between the P50 and P1 dose pools as well as between the different parasite strains.

### Sexual stage efficacy

**Fig. 6:**
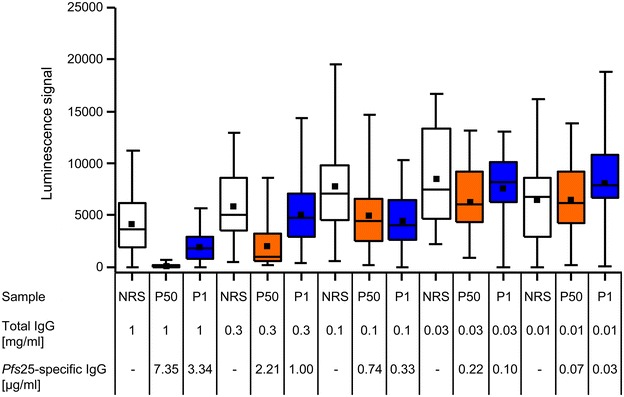
Confirmation of the transmission-blocking activity of antibodies induced by VAMAX-Mix immunization by standard membrane feeding assay (SMFA). The SMFA was performed with total IgG purified from neutral rabbit serum (NRS, control, *white*), P1 (1 µg antigen dose, *blue*) and P50 (50 µg antigen dose, *orange*) at 1, 0.3, 0.1, 0.03 and 0.01 mg/ml, and the mean luminescence intensity values for 24 individual mosquitoes from a single experimental feeds are shown (the* box* indicate the 25th and 75th percentiles whereas the* whiskers* indicate minimum and maximum luciferase values). The transmission-blocking activity is expressed by comparing the mean luciferase activity of P1 and P50 with the corresponding luciferase activity in the NRS control group. The dose-dependency of the transmission-blocking activity was used to determine the IC_50_ values for total IgG as well as *Pfs*25-specific antibodies and the results are summarized in Table 3

The potential transmission-blocking activity of the VAMAX 1, 2 and 6 specific rabbit immune IgG was analysed by SMFA. The transmission-blocking activity of sera derived from the P1 and P50 dose pools correlated with the concentration of *Pfs*25-specific IgG (Fig. 6). As shown in Table 3, the estimated IC_50_ did not deviate significantly between the P1 and P50 dose pools (1.409 and 1.049 µg/ml, respectively, for *Pfs*25-specific IgG).

## Discussion

After the predominantly disappointing results achieved with many single-component malaria vaccine candidates in clinical trials, the concept of combining different *P. falciparum* antigens to improve, immunogenicity, efficacy and strain coverage has received greater attention [38, 39]. The authors of this article are following different approaches towards the development of a multi-stage multi-component malaria vaccine cocktail. In this context, a vaccine cocktail was developed [20] comprising three recombinant fusion proteins based on the three diversity-covering (DiCo) variants of the *P. falciparum* apical membrane antigen *Pf*AMA1 developed by Remarque et al. [13], each featuring *Pfs*25 fused to the C-terminus of the *Pf*AMA1_DiCo variant, and additionally the TSR-domain of *Pf*CSP (VAMAX 1), the 19 kDa C-terminal fragment of *Pf*MSP1 (VAMAX 2) or *Pf*CelTOS (VAMAX 4). A further approach based on a cocktail of VAMAX 1, 2 and 4 used a hyper-immunization protocol to induce high immune IgG titers, to generally confirm and dissect the multi-stage in vitro efficacy of the fusion antigen cocktail. In the current study, the dose-dependency of antigen-specific titers and in vitro efficacy was investigated based on a more realistic immunization scheme and a human-compatible adjuvant. Because proteolytic cleavage of the C-terminal *Pf*CelTOS domain in the DiCo3-based VAMAX4 was previously observed, a modified variant (VAMAX 6) was used in this study, in which *Pf*CelTOS was replaced with the Q5A epitope from the blood-stage antigen *Pf*RH5.

All three fusion proteins (VAMAX 1, 2 and 6) were produced by *Pichia* *pastoris* fed-batch fermentation and a purity of >95 % was achieved after IMAC and SEC for all three proteins. The observed yields (VAMAX 1 10 µg/ml, VAMAX 2 13 µg/ml, VAMAX 6 4 µg/ml, calculated after purification given as µg fusion protein/ml culture supernatant) were within the previously observed range for the production and purification of the VAMAX 1, 2 and four variants [20] but lower than the yields originally reported for *Pf*AMA1 (120 µg/ml, after purification) [40]. Lower expression levels may result either from intrinsic features of the fusion partner, or from the increased size of the fusion protein for different *Pf*AMA1_DiCo fusion proteins where yield was inversely related to the protein size [41]. The integrity and conformation of the recombinant proteins was investigated by LDS-PAGE, immunoblot with antigen-domain-specific antibodies, and mass spectrometry, confirming the presence of intact, full-size proteins. There was no indication of proteolytic cleavage, previously reported for the VAMAX four component in which most of the C-terminal *Pf*CelTos domain was found to be cleaved off from the fusion protein [20].

A one prime/two boost immunization scheme (protein in adjuvant formulation) of groups of four rabbits with four different doses (0.1, 1, 10 and 50 µg) of an equimolar mixture of the three recombinant fusion proteins (VAMAX 1, 2 and 6), using the human-compatible adjuvant Alhydrogel®, yielded a low titer (3.8 × 10^4^) for the 0.1-µg dose, and reasonable titers (5.6 × 10^5^–2.5 × 10^6^) for the three higher doses with no differences between the 1 and 10 µg doses. This agrees with the results of a recent mouse immunization study using 0.01, 0.03, 0.1, 0.3 and 1 µg doses of *Pf*AMA1_DiCo mix formulated in alum (Rehydragel®) which also showed clear dose-dependent seroconversion (Faber et al., pers. comm..). In contrast, no significant differences in total IgG levels were observed for the four different doses, indicating that the induced total IgG is adjuvant-dependent rather than antigen-dose-dependent in the investigated context.

Specific IgG concentrations were determined for all included antigen domains except the Q5A epitope, where no reactivity could be shown in ELISA. Even though Faber et al. have demonstrated that fusing smaller antigen domains like *Pf*MSP1_19 to *Pf*AMA1_DiCo could improve immune responses against a smaller domain that suffers from low immunogenicity when used alone [41], Q5A might be too small to sufficiently benefit from this effect in the context of the VAMAX6 fusion protein.

For the blood stage it was possible to attribute the growth inhibition activity (measured by GIA) exclusively to *Pf*AMA1-specific antibodies because the addition of an excess of *Pf*AMA1_DiCo mix completely abolished parasite growth inhibition in a reversal of growth inhibition assay. In this context, neither the *Pf*MSP1_19 specific antibodies (P1 = 14 µg/ml and P50 = 43 µg/ml *Pf*MSP1-19 in 10 mg/ml total IgG, respectively) nor any potential *Pf*RH5_Q5A-specific antibodies (not determined) contributed to the inhibition of growth. As previously suggested [20], this phenomenon may reflect the presence of *Pf*MSP1_19-specific and *Pf*RH5_Q5A-specific antibodies that are below the concentration required for the reliable determination of growth inhibition in GIAs (>100 µg/ml for *Pf*MSP1_19-specific antibodies [41] and >30 µg/ml for *Pf*RH5_Q5A-specific antibodies [21]).

To investigate whether the in vitro protective efficacy of the induced immune responses is directly proportional to the amount of relevant, component-specific IgG, CFCA analysis was carried out using the different components (*Pf*CSP_TSR, *Pf*AMA1_DiCo mix, *Pf*MSP1_19 and *Pfs*25). The estimated in vitro IC_50_ values observed for the pre-erythrocytic-stage antigen *Pf*CSP_TSR (12 µg/ml for P50), the blood-stage antigen *Pf*AMA1_DiCo mix (120–140 µg/ml for P50) and the sexual-stage antigen *Pfs*25 (1.0 µg/ml for P50) were in the same range as reported in other studies featuring or including these antigens in comparable assays [13, 20, 29, 42].

The correlation of these antibody concentrations with the estimated IC_50_ values observed in the corresponding stage-specific parasite-inhibition assays confirmed the absence of qualitative differences in terms of in vitro efficacy between antigen-specific IgG induced by different doses of the antigen cocktail, and that the efficacy in relation to total IgG is only dose-dependent in this context. Comparable IC_50_ values were also obtained for hyperimmunization with the predecessor cocktail (VAMAX 1, 2 and 4) using a strong lipopolysaccharide-based formulation (Biogenes proprietary adjuvant) [20], which further indicates that maximizing antigen-specific titers should be the main focus of further optimization efforts, preferentially with higher doses >50 µg, which should not be critical, when looking at the antigen doses used within approved complex combination vaccines such as Infanrix hexa® (GSK). Additionally, the potential of other human compatible adjuvants like Motanide ISA720 and AS02 (as shown for an *Pf*AMA1-FVO-based vaccine candidate by Roestenberg et al.) or combinations thereof to significantly increase antibody responses against the VAMAX-mixture could be investigated [28]. Furthermore, alternative particulate presentation formats like virus-like-particle fusions, currently being evaluated in a clinical trial (ClinicalTrials.Gov Id: NCT02013687) with the *P.falciparum* sexual stage antigen *Pfs*25, would open an additional strategy to improve immunogenicity.

**Fig. 7:**
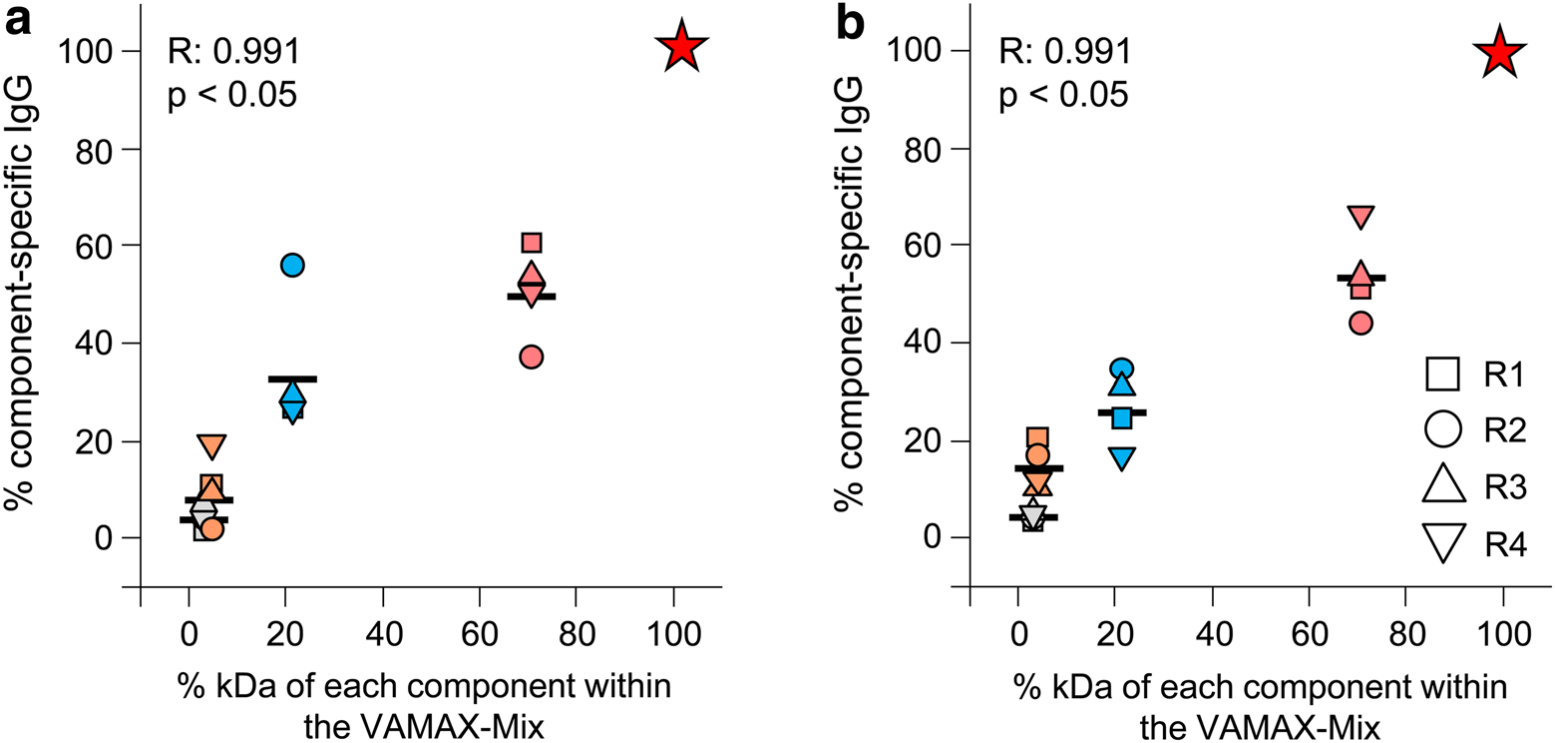
Molecular weight dependency of component-specific reactivity.* Y*-axis: Relative proportion of specific IgG calculated for the five components using the CFCA results of P1 (**a**) and P50 (**b**). The sum of individual component-specific IgG concentrations was set to 100 %.* X*-axis: Relative proportion of molecular weight calculated for the five components. The sum of molecular weights of VAMAX 1, VAMAX 2 and VAMAX 6 was set to 100 %. Reference points: 0/0 % (not depicted) and 100/100 % (*red star*), *Pf*AMA1_DiCo mix (*red*), *Pfs*25 (*blue*), *Pf*MSP1_19 (*orange*) and *Pf*CSP_TSR (*light gray*). Each symbol represents the value for each individual rabbit (R1, R2, R3 and R4). The corresponding geometric means are included as horizontal *black lines*. For detailed statistical analysis refer to the “Methods” section

The antigen component-specific IgG amounts showed an almost linear correlation with the quantitative representation of the corresponding component within the context of the mixture (Fig. 7), agreeing with the results presented in the previous study [20], and further emphasizing the potential to explore this correlation for the “fine tuning” of stage-specific functionality according to the strongly-biased IC_50_ requirements for parasite growth inhibition (at least based on in vitro efficacy in rabbits) in the context of malaria vaccine development. Small domains (e.g. *Pf*MSP1-19) and promising inhibitory epitopes (e.g. *Pf*RH_5Q5A) are most likely to require multivalent presentation, when used in combination with more and/larger antigens (e.g. *Pf*AMA1).

## Conclusions

This study demonstrates that the amounts of vaccine-specific antibodies induced following the immunization of rabbits with four different doses (0.1, 1, 10 and 50 µg) strongly depend on antigen dose when using a human-compatible adjuvant (Alhydrogel®) and vaccination scheme (one prime/two boosts). A comparison of the results with those obtained in an earlier study based on a hyper-immunization regime, in which higher levels of antigen-specific IgG were observed, suggests that there is significant need for improvement to generate a higher immune response and match efficacy requirements, especially for a *Pf*AMA1-based blood stage vaccine, by using higher doses, better adjuvants and/or better formulations. The results also confirm the previously described strong positive correlation between antigen-specific antibody titers and the quantitative representation of the corresponding component within the vaccine mixture, suggesting an overrepresentation of smaller antigens or peptides e.g. by increasing valency or by formation of VLPs would be beneficial.

## Abbreviations

IgG: immunoglobulin G
IC_50_: half maximal inhibitory concentration
*Pf*AMA1: *Plasmodium falciparum* apical membrane antigen 1
*Pf*CSP: *Plasmodium falciparum* circumsporozoite protein
*Pf*MSP: *Plasmodium falciparum* merozoite surface protein
DiCo: diversity covering
TBV: transmission-blocking vaccine
AA: amino acid
AOX1: alcohol oxidase 1
H/D: height/diameter
DO: dissolved oxygen
IMAC: immobilized metal ion affinity chromatography
SEC: size exclusion chromatography
LDS: lithium dodecylsulfate
PAGE: polyacrylamide gel electrophoresis
TSR: thrombospondin-related
MS: mass spectrometry
HPLC: high performance liquid chromatography
ETD: electron-transfer dissociation
ESI: electrospray ionization
CID: collision-induced dissociation
EDTA: ethylenediaminetetraacetic acid
GMP: good manufacturing practice
ELISA: enzyme-linked immunosorbent assay
PBS: phosphate buffered saline
CFCA: calibration-free concentration analysis
EDC-NHS: 1-ethyl-3-(3-dimethylaminopropyl)carbodiimide-N-hydroxysuccinimide
SFMA: standard membrane feeding assay
RBC: red blood cell
SGM: sporozoite gliding motility
SILSD: sporozoite invasion and liver stage development
DAPI: 4,6-diamidino-2-phenylindole
MALDI: matrix-assisted laser desorption ionization
TOF: time of flight
